# Co-segregation of *WFS1* p.Gly494Ser variant with recurrent vasovagal syncope in a multigenerational family: a candidate susceptibility variant needing independent validation

**DOI:** 10.3389/fped.2026.1841869

**Published:** 2026-09-03

**Authors:** Xinxin Guo, Jie Chen, Xiaoli Wang, Xi Zheng, Fang Yang

**Affiliations:** Department of Pediatrics, Fuzhou University Affiliated Provincial Hospital, Fuzhou, Fujian, China

**Keywords:** autonomic dysfunction, genetic mutation, vasovagal syncope, *WFS1* gene, whole-exome sequencing

## Abstract

**Introduction:**

Vasovagal syncope (VVS) is the most prevalent subtype of reflex syncope. However, the genetic etiology underlying familial recurrent VVS has not been fully elucidated.

**Methods:**

We recruited a multigenerational Chinese pedigree with three-generation recurrent early-onset VVS. Whole-exome sequencing was performed to screen pathogenic variants. Variant interpretation was conducted following the ACMG-AMP 2015 criteria, and multiple in-silico prediction tools were applied to evaluate variant pathogenicity. Clinical laboratory indicators of affected family members were collected and analyzed.

**Results:**

The heterozygous WFS1 c.1480G>A (p.Gly494Ser) variant was identified and found to co-segregate with the syncope phenotype within this family. This variant is rare in public population databases and predicted to be potentially damaging to wolframin protein structure. It was classified as a Variant of Uncertain Significance (VUS) with moderate-strength evidence of PM2 and PP3. Concurrent hyperinsulinemia was detected in affected individuals, which may be confounded by obesity-related insulin resistance.

**Discussion:**

Our results hypothesize that WFS1 p.Gly494Ser may serve as a candidate susceptibility variant for VVS. This finding broadens the phenotypic spectrum of heterozygous WFS1 variants and provides preliminary evidence linking endoplasmic-reticulum-related and autonomic regulatory pathways to VVS predisposition. Further validation using large independent cohorts and comprehensive cellular functional experiments is required to confirm the causal role of this variant in VVS.

## 1 Introduction

Vasovagal syncope (VVS) is the leading cause of transient loss of consciousness in children and adolescents, arising from transient autonomic-cardiovascular dysregulation marked by inappropriate vasodilation and/or bradycardia, often triggered by prolonged standing, emotional stress, pain or dehydration. Though the Bezold-Jarisch reflex is thought to underpin VVS, its multifactorial pathogenesis remains incompletely defined (1). Twin studies support a heritable contribution to VVS (2); yet heritability alone cannot account for familial clustering, suggesting unvalidated genetic predisposition as a potential risk factor.

**Figure 1:**
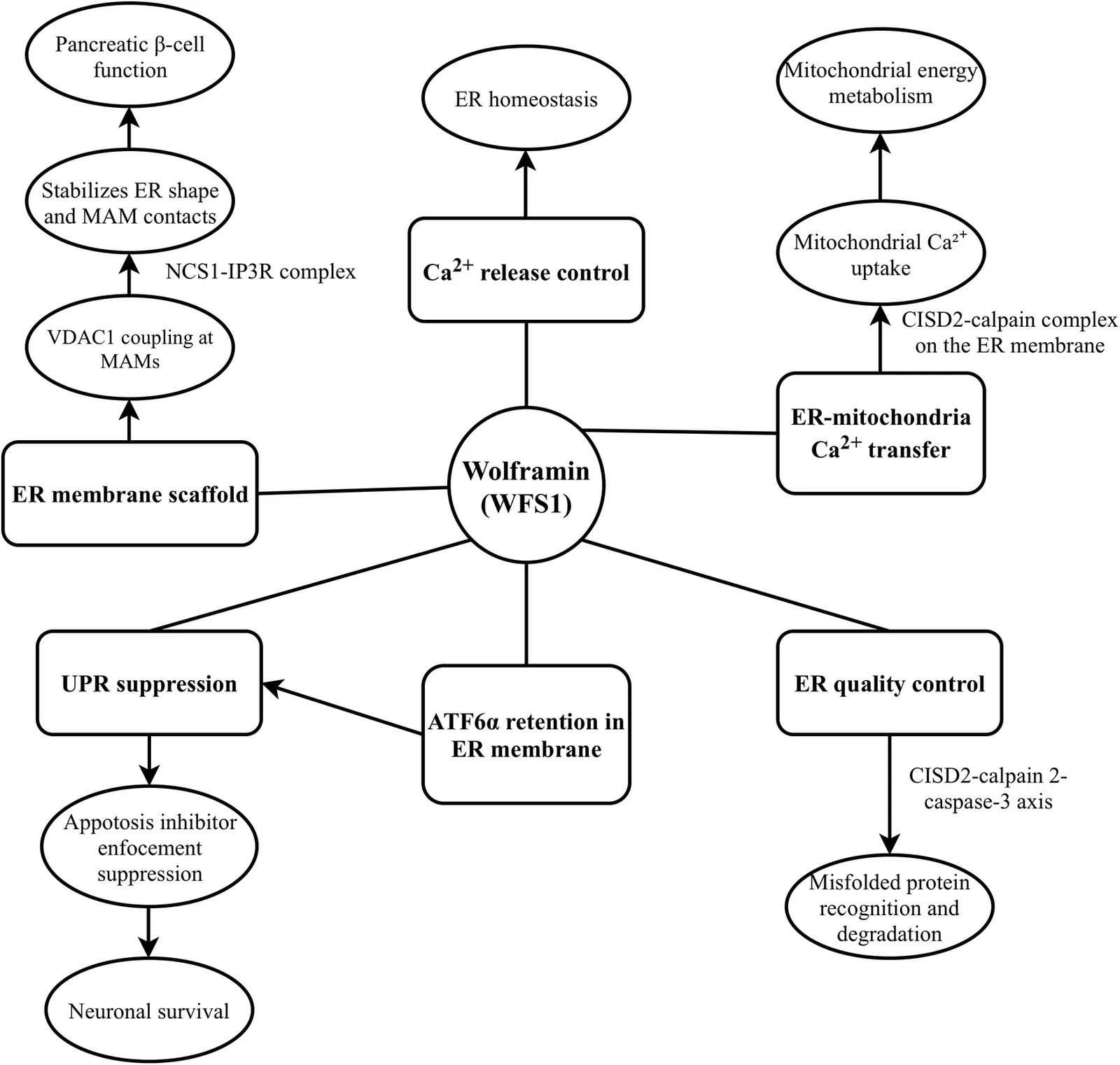
Functional schematic of wolframin (*WFS1* protein) illustrating its primary molecular regulatory roles and downstream biological outcomes. Centered on wolframin, this schematic outlines its five core functional branches: (1) acting as an ER membrane scaffold to interact with VDAC1 and regulate the NCS1-IP3R complex for structural stabilization of ER and MAM compartments and maintenance of pancreatic β-cell function; (2) controlling ER ${\mathrm{Ca}}^{2+}$ release to preserve ER homeostasis; (3) sequestering ATF6α in the ER membrane to suppress UPR, strengthen anti-apoptotic effects and ensure neuronal survival; (4) conducting ER quality control via the CISD2-calpain 2-caspase-3 axis to eliminate misfolded proteins; (5) mediating ER-mitochondria ${\mathrm{Ca}}^{2+}$ translocation through the ER-membrane CISD2-calpain complex to boost mitochondrial ${\mathrm{Ca}}^{2+}$ uptake and energy metabolism. ER, endoplasmic reticulum; MAMs, mitochondria-associated membranes; UPR, unfolded protein response.

Located at chromosome 4p16.1, *WFS1* encodes the ER-resident transmembrane protein wolframin, which maintains ER homeostasis, intracellular calcium dynamics and ER-stress responses (Figure 1) (3). Biallelic *WFS1* pathogenic variants cause classic Wolfram-syndrome-spectrum disorders (4), whereas heterozygous variants produce diverse mild phenotypes including non-syndromic hearing loss and neuroendocrine abnormalities (5), indicating wolframin functions extend beyond canonical Wolfram-syndrome manifestations.

Wolframin is highly expressed in neuronal and neuroendocrine tissues and participates in autonomic and endocrine signaling (6, 7). Given the central role of autonomic reflex dysfunction in VVS pathophysiology, variants perturbing neuronal signaling, calcium homeostasis and cellular stress may alter VVS susceptibility. However, the association between *WFS1* variants and VVS remains uninvestigated.

Here, we studied a multigenerational family with recurrent VVS among maternal relatives. Whole-exome sequencing and familial co-segregation analysis uncovered a heterozygous *WFS1* missense variant detected in all affected but no unaffected family members. Bioinformatic analyses were applied to predict its structural impact. Combining co-segregation evidence, population genetics and in silico structural modelling, we propose *WFS1* c.1480G>A as a plausible candidate susceptibility variant for familial VVS and provide testable hypotheses for future mechanistic research. No cellular functional assays were performed in this study.

## 2 Materials and methods

### 2.1 Study design and participants

This family-based clinical and genetic study aimed to explore whether *WFS1* variants confer susceptibility to VVS. The proband diagnosed with VVS was recruited from the Department of Pediatrics; available first- and second-degree relatives were enrolled for clinical and genetic assessment.

Clinical data were collected from medical records and follow-up visits, and a pedigree was constructed via family interviews. The study was approved by the institutional ethics committee (K2023-09-008). Written informed consent was obtained from all adult participants and guardians of minor subjects prior to enrollment. Partial participants declined full panels of invasive or prolonged examinations (HUTT, OGTT, brain MRI) owing to age, personal willingness or clinical necessity; individual test information is summarized in Table 1.

**Table 1:** Clinical and genetic characteristics of enrolled family members.

Characteristic	Proband (IV-1)	Mother (III-1)	Grandmother (II-4)	Father (III-2)	Half-sister (IV-2)	Greatuncle (II-2)	Cousin aunt (III-3)
Age (years)	13	37	67	40	2.5	71	42
Sex	Male	Female	Female	Male	Female	Male	Female
*WFS1* Genotype	Het c.1480G>A (p.Gly494Ser)	Het c.1480G>A (p.Gly494Ser)	Het c.1480G>A (p.Gly494Ser)	Wild-type	Wild-type	Wild-type	Wild-type
Syncope/presyncope history	Recurrent syncope (2 episodes/5 months)	Recurrent presyncope + syncope	Lifelong recurrent syncope (adolescence–55y)	–	–	–	–
Age of symptom onset	12 y	10 y	14 y	–	–	–	–
HUTT result	Positive (mixed-type VVS)	Positive (mixed-type VVS)	NP	NP	NP	NP	NP
Episode-related BP/HR	BP 76/40 mmHg, HR 53 bpm	BP 75/36 mmHg, HR 55 bpm	Historical BP 80/50 mmHg, HR 52 bpm	–	–	–	–
OGTT + insulin release test	Completed, hyperinsulinemia confirmed	Completed, hyperinsulinemia confirmed	NP	NP	NP	NP	NP
Diabetes status	No	No	Type 2 DM (onset 62 y)	No	No	No	No
Ophthalmologic findings	Normal (no optic atrophy/visual loss)	Normal	Normal	Normal	Normal	Normal	Normal
Audiological findings	Normal (no sensorineural hearing loss)	Normal	Normal	Normal	Normal	Normal	Normal
Other key clinical features	Obesity, mild neck hyperpigmentation	Normal BMI, no pigmentation	Controlled age-related hypertension	Unremarkable exam	Unremarkable exam	Mild osteoarthritis	Unremarkable exam
Ambulatory blood pressure	Normal	Normal	Hypertension	NP	NP	NP	NP
Echocardiography	Normal	Normal	Mild diastolic dysfunction	NP	NP	NP	NP
Electroencephalography	Normal	Normal	Normal	NP	NP	NP	NP
Abdominal ultrasonography	Normal	Normal	Normal	NP	NP	NP	NP
Brain magnetic resonance imaging	Normal	Normal	NP	NP	NP	NP	NP

1. All clinical data are extracted from medical records and standardized clinical assessments.

2. *WFS1* genotyping was performed via Sanger sequencing for all participants.

3. VVS, vasovagal syncope; HUTT, head-up tilt test; BP, blood pressure; HR, heart rate; OGTT, oral glucose tolerance test; DM, diabetes mellitus; NP, Not performed.

4. Dash (–) indicates no relevant clinical manifestations.

### 2.2 Clinical evaluation

All participants received standardized assessments including medical history inquiry and physical examination. Examinations contained OGTT, insulin release assay, 24-h ambulatory blood pressure, Holter ECG, electroencephalography, echocardiography, brain MRI and head-up tilt testing (HUTT). VVS diagnosis was confirmed by typical manifestations and positive HUTT results after excluding cardiac, neurological and metabolic causes of transient unconsciousness. Clinical management and follow-up outcomes were documented accordingly.

### 2.3 Whole-exome sequencing and variant filtering

Peripheral blood (5 mL) from the proband, parents and four additional family members was collected for genomic DNA extraction and whole-exome sequencing (WES). Raw sequencing data underwent quality control, alignment, variant calling and annotation. We screened rare coding and splice-site variants predicted to disrupt protein structure or function.

Variants with minor allele frequency >5% in ExAC, 1,000 Genomes and gnomAD were excluded. Remaining variants were annotated via dbSNP, OMIM, HGMD and ClinVar. Candidate variants were prioritized by rarity, predicted functional damage, familial co-segregation and biological relevance to autonomic and syncope-related pathways. Variants lacking co-segregation were discarded. Sanger sequencing validated candidate variants, followed by co-segregation analysis within the pedigree.

### 2.4 Evolutionary conservation and pathogenicity prediction

The reference sequence of *WFS1* was obtained from the National Center for Biotechnology Information (NCBI) database. Multiple sequence alignment of *WFS1* protein sequences from different species was performed to evaluate evolutionary conservation at the affected amino acid residue.

The potential pathogenicity of the identified missense variant was assessed using PolyPhen-2 (http://genetics.bwh.harvard.edu/pph2/index.shtml), a computational prediction tool designed to estimate the impact of amino acid substitutions on protein structure and function.

### 2.5 Secondary and tertiary structure analysis

Secondary structure prediction of wild-type and mutant *WFS1* proteins was performed using the Self-Optimized Prediction Method with Alignment (SOPMA) online platform (https://npsa-prabi.ibcp.fr/cgi-bin/npsa_automat.pl?page=/NPSA/npsa_sopma.html). The predicted proportions of α-helices, β-sheets, β-turns, and random coils were compared to evaluate potential structural alterations associated with the identified variant.

Three-dimensional structural models of wild-type and mutant *WFS1* proteins were generated using AlphaFold-based prediction platforms (https://alphafold.ebi.ac.uk). Comparative structural analysis was performed to assess potential conformational changes resulting from the amino acid substitution.

### 2.6 Statistical considerations

Given the exploratory nature of this study and the limited number of individuals available within a single pedigree, formal statistical association analysis was not performed. Instead, evidence supporting the potential relevance of the identified variant was evaluated through familial co-segregation analysis, population frequency assessment, evolutionary conservation, pathogenicity prediction, and structural modeling. The findings should therefore be considered preliminary and hypothesis-generating, requiring validation in larger cohorts and functional studies.

## 3 Results

### 3.1 Clinical findings

The proband was a 13-year-old boy presenting with two transient visual blackout episodes within five months. The first attack occurred while awaiting gastrointestinal endoscopy anesthesia, featuring blurred vision, dizziness, pallor and diaphoresis without complete loss of consciousness; symptoms resolved rapidly after intravenous fluid infusion. The second episode happened post-showering, accompanied by hypotension (84/53 mmHg) and bradycardia (53 beats/min), with spontaneous recovery following rest and oral glucose intake.

During hospitalization, the proband experienced a typical syncopal-like episode triggered by fasting venipuncture, characterized by visual blackout, dizziness, pallor, diaphoresis, hypotension and bradycardia, with normal capillary glucose. Routine laboratory tests including complete blood count, biochemistry, thyroid function, cortisol, ACTH, glycated hemoglobin, catecholamines and islet autoantibodies were unremarkable. Further work-up (24 h ambulatory blood pressure, Holter, EEG, echocardiography, abdominal ultrasound, brain MRI, ophthalmological and audiological assessment) revealed no clinically significant organic abnormalities. OGTT and insulin-release testing confirmed hyperinsulinemia (Table 2).

**Table 2:** Oral glucose tolerance test (OGTT) and insulin release test results.

Variables	Test time points						
	0 h	0.5 h	1 h	2 h	3 h	4 h	5 h
Proband							
Blood Glucose (mmol/L)	4.91	7.98	9.41	6.19	6.78	3.64	3.28
Insulin (mU/L)	14.7	374.5	725.5	198.2	264.8	42.4	21.1
Proband’s mother							
Blood Glucose (mmol/L)	5.31	8.12	9.98	7.18	7.06	5.62	4.30
Insulin (mU/L)	16.8	380.6	746.5	200.3	196.2	56.7	25.4

1. All values are presented as raw measurement results.

2. In healthy individuals, fasting insulin levels range from 5 to 20 mU/L. After oral glucose administration, insulin levels typically peak at 30 to 60 min, reaching approximately 5 to 10 times the fasting level (50 to 100 mU/L).

3. Subsequently, insulin levels gradually decline and generally return to fasting levels within 3 h.

**Table 3:** Variant filtering pipeline for whole-exome sequencing (WES) data.

Filter step	Remaining variants	Filtered out variants	Detailed filtering criteria
Raw called variants from whole-exome sequencing (WES)	124,872	0	Initial variant calling output from GATK pipeline after alignment of sequencing reads of 7 family members (proband, parents, 4 additional relatives)
Remove low-quality/low-depth variants (read depth <10×, mapping quality <30, base quality <20)	89,315	35,557	Exclude variants with insufficient sequencing coverage or poor alignment/base calling quality
Remove synonymous variants and non-coding/non-splice-site intergenic/intronic variants	41,628	47,687	Retain only exonic variants and canonical splice-site variants; discard silent synonymous substitutions and non-functional non-coding variants
MAF filtering (exclude variants with MAF >5% in ExAC, 1000 Genomes Project, gnomAD)	3,791	37,837	Remove common population polymorphisms with minor allele frequency exceeding 5% across three major public population databases
Remove variants with no predicted functional impact (in silico neutral predictions: REVEL <0.5, PolyPhen-2 benign, SIFT tolerated)	942	2,849	Keep variants predicted to disrupt protein structure/function by multiple bioinformatic tools
Remove variants without co-segregation with disease phenotype in the pedigree	1	941	Exclude variants that do not segregate with clinical manifestations across the enrolled family; retain only the *WFS1* candidate variant
Final candidate variant for Sanger validation & co-segregation analysis	1	0	Single variant subjected to orthogonal Sanger sequencing verification

1. All variant counts are presented as absolute numbers.

2. The filtering pipeline follows standard WES variant prioritization workflow for monogenic disease candidate variant identification.

3. MAF, Minor Allele Frequency; WES, Whole-Exome Sequencing; GATK, Genome Analysis Toolkit.

**Table 4:** Annotation details of the *WFS1* candidate variant.

Variant annotation item	Variant value
Gene	*WFS1*
Transcript ID	NM_006005.3
Chromosomal position (GRCh37/hg19)	chr4:6303002
Nucleotide change	c.1480G>A
Amino acid change	p.Gly494Ser
Variant type	Missense variant (exonic)
Maximum population MAF (gnomAD/ESP/1000G)	0.0004
REVEL score	0.862
Genotype (Proband)	Heterozygous (Het)
Genotype (Mother)	Heterozygous (Het)
Genotype (Father/Siblings)	Wild-type (WT, unaffected)
ACMG classification	Variant of uncertain significance (VUS, PP3_Moderate)

1. The variant is located in the coding region of the *WFS1* gene, which is associated with Wolfram syndrome.

2. MAF, Minor Allele Frequency.

3. ACMG classification follows the 2015 American College of Medical Genetics and Genomics standards for variant interpretation.

Recurrent syncope/presyncope could be provoked by walking, emotional stress, food intake or cycling. Symptomatic episodes were accompanied by reduced blood pressure and heart rate, whereas peripheral glucose remained normal. Positive head-up tilt-table test supported mixed-type VVS. The patient was diagnosed with mixed-type VVS complicated by hyperinsulinemia. No further syncopal events occurred after oral midodrine treatment. Physical examination showed stable vital signs and obesity; mild neck hyperpigmentation without typical acanthosis nigricans. Cardiac, respiratory, abdominal and neurological examinations were unremarkable.

### 3.2 Genetic findings

Whole-exome sequencing identified a heterozygous *WFS1* missense variant $NM_{0}06005.3\;:\;c.1480G>A$ (p.Gly494Ser) in the proband. This variant exhibited a maximum allele frequency of 0.0004 across public population databases (gnomAD, ExAC, 1,000 Genomes), far below thresholds for benign variants, satisfying ACMG-2015 PM2 evidence. Its extreme rarity excludes common neutral polymorphisms, yet no prior studies have linked this specific variant to VVS, and pathogenic potential remains unproven.

**Figure 2:**
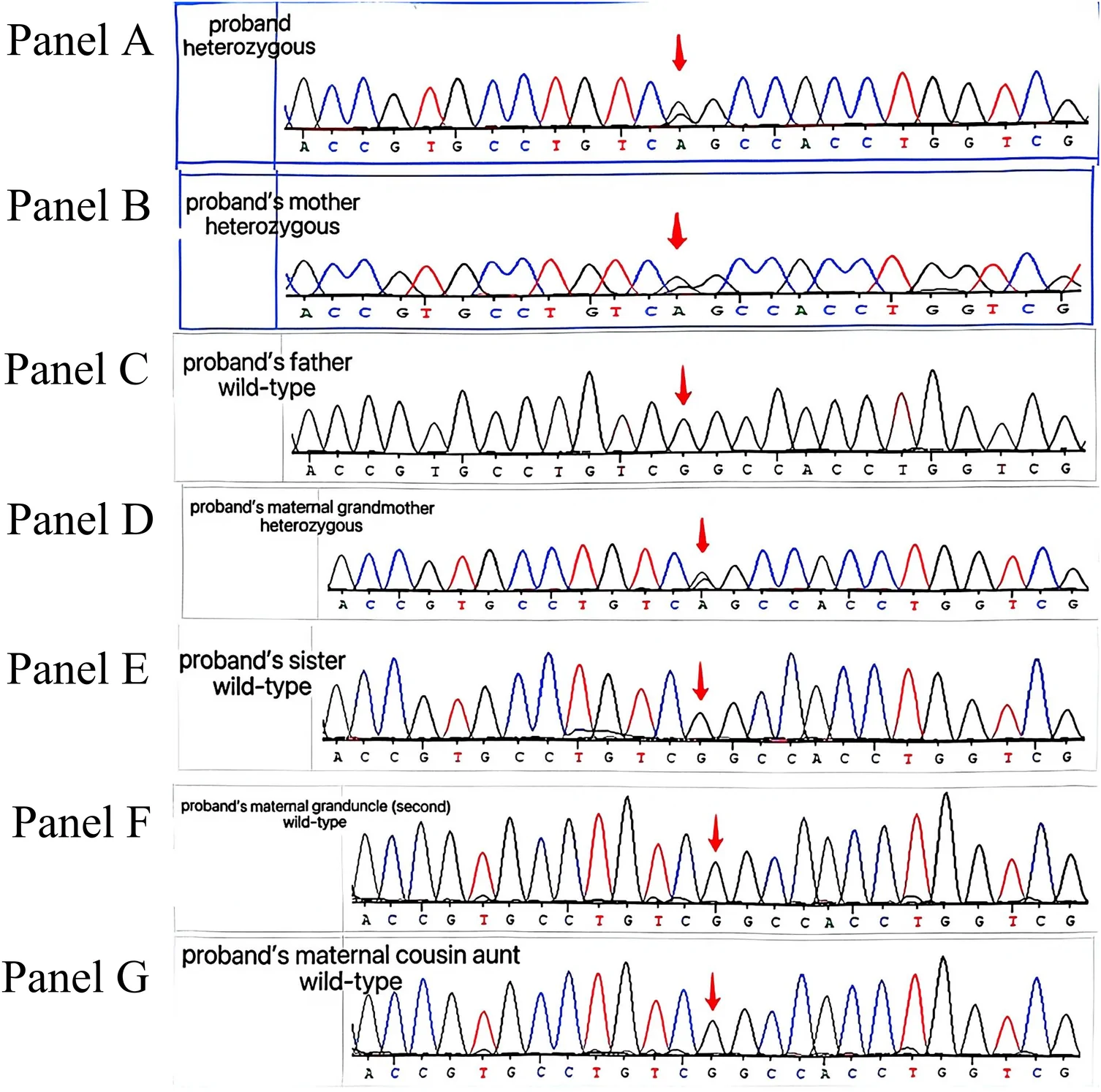
Sanger sequencing of the *WFS1* gene in the proband (Panel **A**), mother (Panel **B**), father (Panel **C**), maternal grandmother (Panel **D**), maternal half-sister (Panel **E**), maternal greatuncle (Panel **F**), and proband’s maternal cousin aunt (Panel **G**). The proband carries the heterozygous *WFS1* variant NM_006005.3:c.1480G>A. The figure shows forward Sanger sequencing chromatograms, and the arrow indicates the variant position.

Sanger sequencing validated this variant in the proband, the proband’s mother and the proband’s maternal grandmother. It was absent in the proband’s father, the proband’s maternal half-sister and other tested unaffected relatives. The three-generation segregation pattern was compatible with autosomal-dominant inheritance (Figure 2).

### 3.3 Genotype–phenotype correlation within the family

Clinical and genetic data were available for seven family members across three generations. The proband, the proband’s mother and the proband’s maternal grandmother all suffered from recurrent VVS-related episodes starting in childhood or adolescence, with decreased blood pressure-heart rate and normal peripheral glucose during attacks. Hyperinsulinemia was documented in both the proband and the proband’s mother by OGTT/insulin-release tests. The proband’s maternal grandmother had recurrent syncope persisting until approximately 55 years of age and later developed type 2 diabetes mellitus, without available insulin-release data. Three maternal granduncles had type 2 diabetes but no syncope history; their offspring were also free of syncopal manifestations.

**Figure 3:**
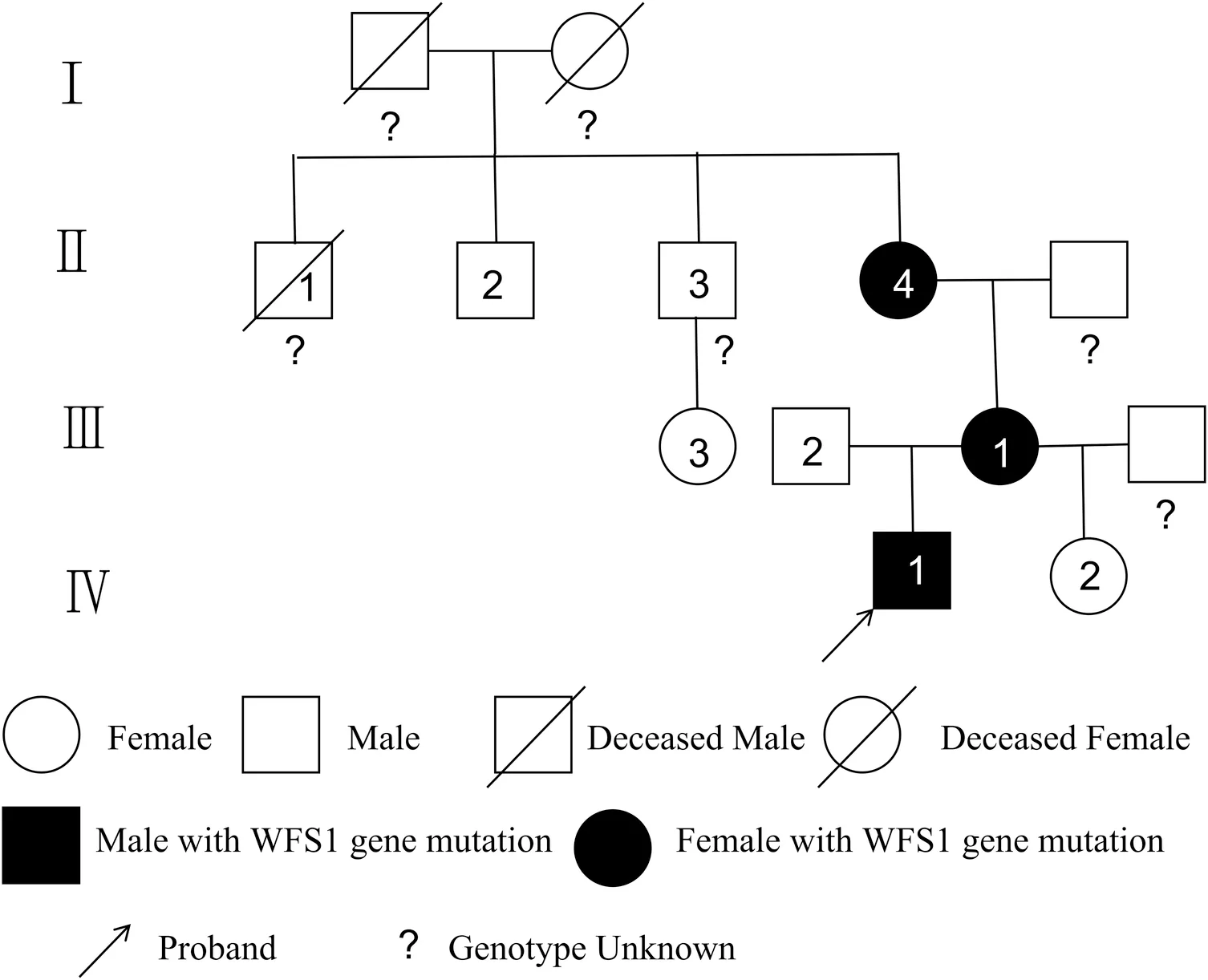
Pedigree of the proband.

The proband’s father and the proband’s maternal half-sister were asymptomatic and variant-negative. Collectively, the variant was detected in all affected carriers and absent among genotyped unaffected relatives, showing familial co-segregation, although causality cannot be established solely by this observation. The pedigree is presented in Figure 3.

### 3.4 Bioinformatics analysis

#### 3.4.1 Evolutionary conservation and pathogenicity prediction

**Figure 4:**
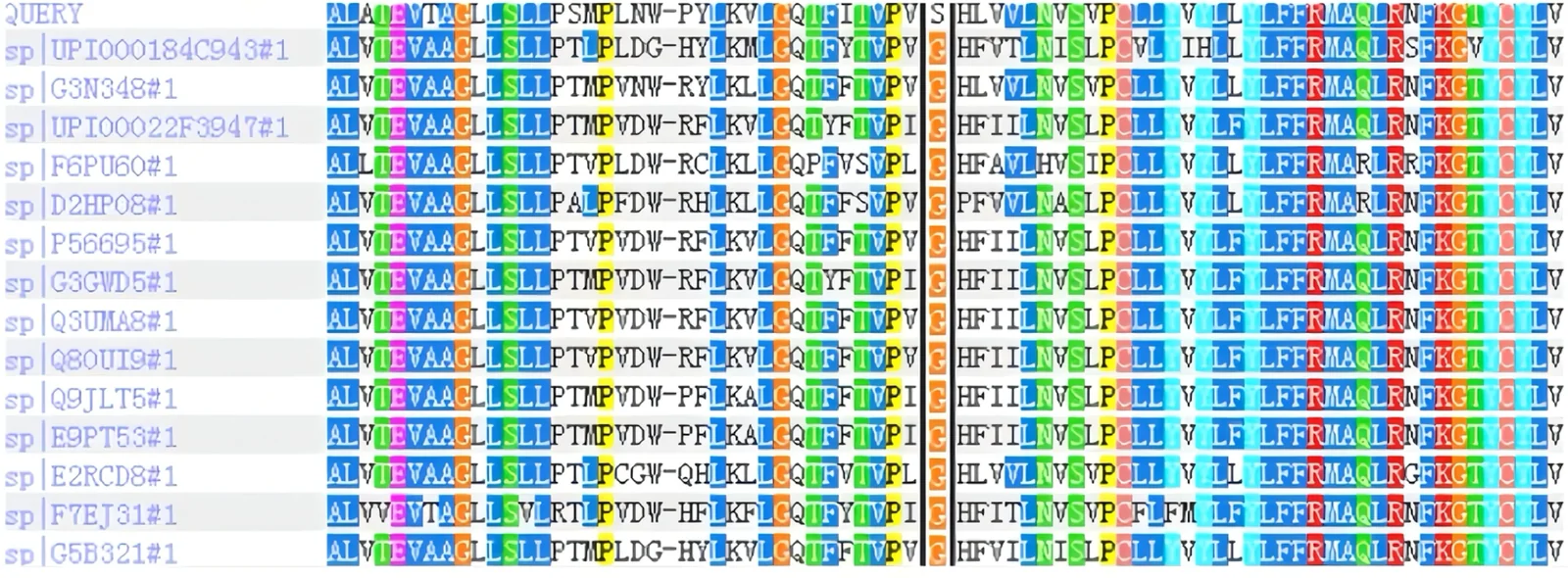
Conservation analysis of the *WFS1* c.1480G>A (p.Gly494Ser) variant site by multi-species sequence alignment.

Multiple-sequence alignment indicated high evolutionary conservation of Gly494 across vertebrates (Figure 4). The substitution c.1480G>A replaces glycine with serine at residue 494. According to gnomAD v3.1.2, the overall MAF was 0.0004 with zero alleles across major subpopulations, fulfilling PM2 (moderate). No corresponding entries were retrieved in ClinVar or HGMD. Concordant damaging predictions from PolyPhen-2, REVEL, SIFT and CADD supported PP3 (moderate). Gly494 locates in the cytosolic juxtamembrane linker C-terminal to transmembrane helix 9 (TM9), a hotspot for reported Wolfram-syndrome-associated missense variants.

Based on ACMG/AMP-2015 criteria (PM2+PP3 moderate), this variant was classified as Variant of Uncertain Significance (VUS). Extreme population rarity argues against common neutral polymorphism but does not prove pathogenicity. Owing to the lack of cellular functional validation and independent cohort replication, this variant is interpreted as a plausible candidate susceptibility variant for familial VVS rather than a proven pathogenic mutation.

#### 3.4.2 Secondary structure analysis

**Figure 5:**
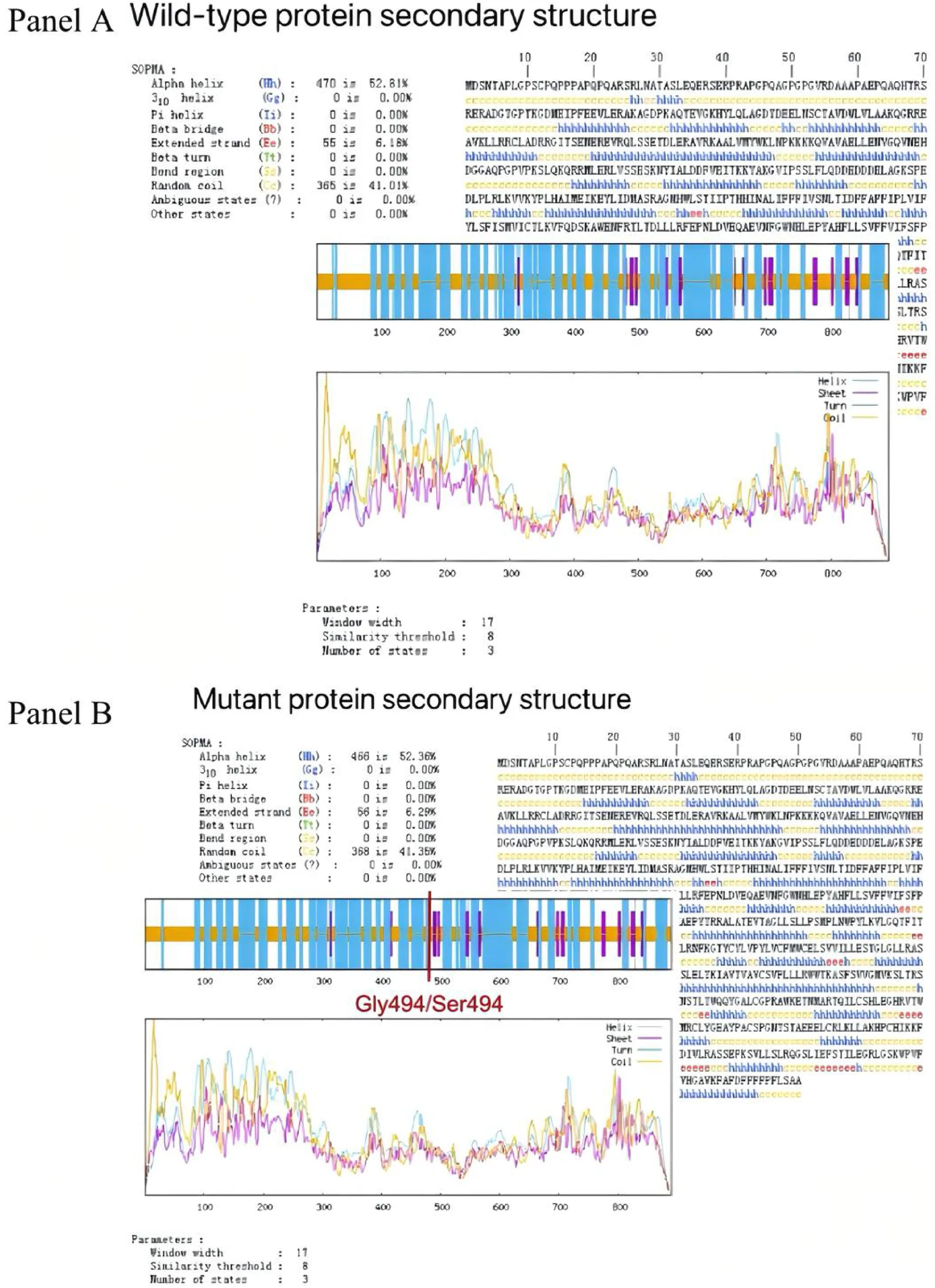
Predicted secondary structures of the wild-type and mutant *WFS1* proteins (Panel **A**: wild-type; Panel **B**: p.Gly494Ser variant).

Topological annotation from UniProt confirmed Gly494 resides within the flexible cytosolic juxtamembrane segment downstream of TM9 (Figure 5), exposed to cytosol. SOPMA secondary-structure prediction suggested a modest reduction in α-helical content with increased random coils for p.Gly494Ser. We note that SOPMA delivers sequence-dependent heuristic predictions with limited reliability for inferring in-vivo functional defects of ER-resident transmembrane proteins.

#### 3.4.3 Tertiary structure modeling

**Figure 6:**
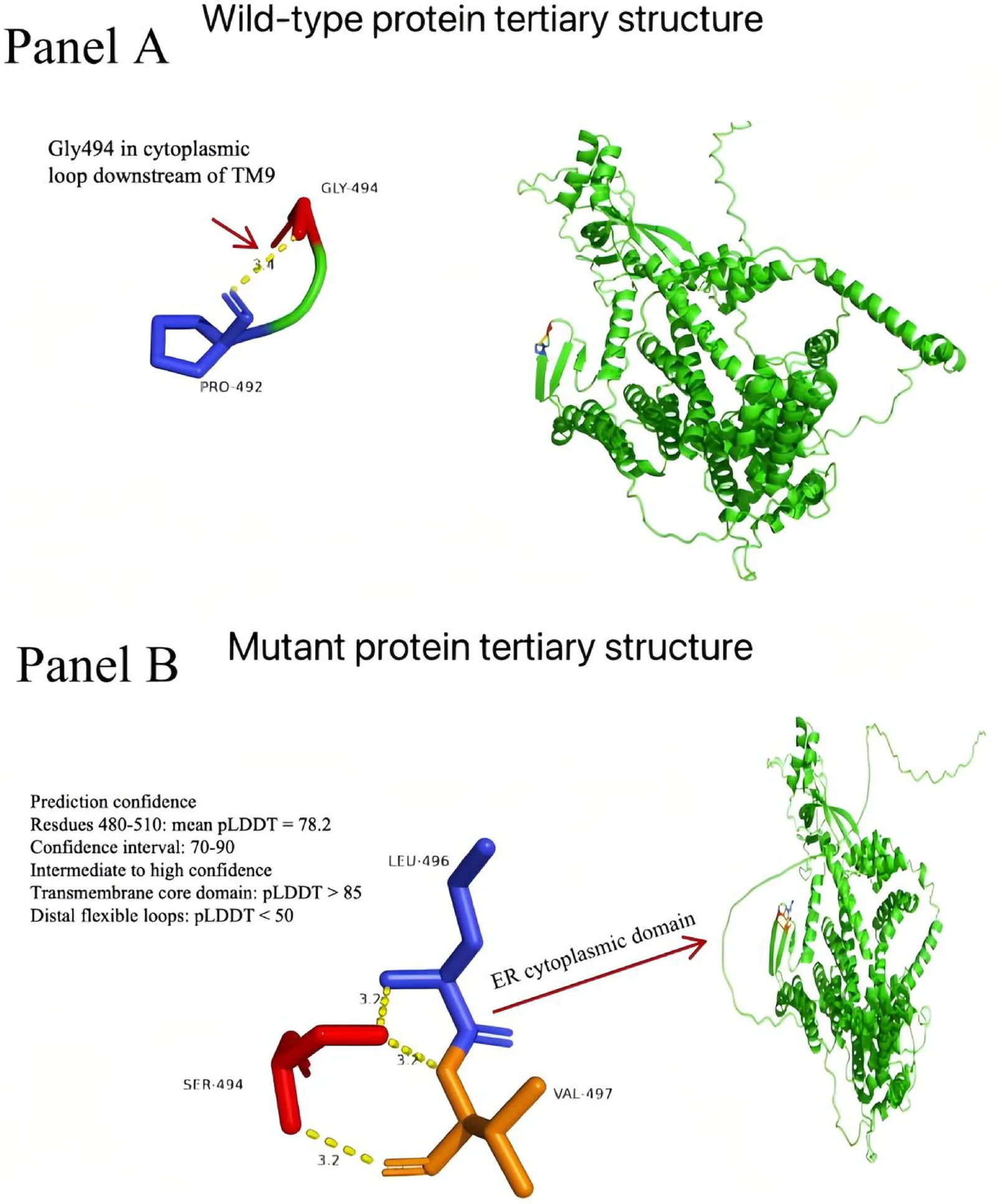
Predicted tertiary structures of the wild-type and mutant *WFS1* proteins (Panel **A**: wild-type; Panel **B**: p.Gly494Ser variant).

AlphaFold2 (v2.3) structural simulation predicted subtle local spatial rearrangement at residue 494 and potential de-novo hydrogen-bond formation in the mutant model (Figure 6). The pLDDT score indicated moderate-high confidence for this juxtamembrane cytosolic segment. Importantly, AlphaFold cannot recapitulate lipid-bilayer embedding, ER-membrane microenvironment or native post-translational modifications. All conformational alterations remain purely in-silico theoretical observations; biological consequences await experimental validation.

## 4 Discussion

Vasovagal syncope (VVS) is the most common form of neurally mediated syncope in children and adolescents and remains a major cause of recurrent transient loss of consciousness in this age group (8). Although considerable progress has been made in understanding its physiological basis, the mechanisms underlying individual susceptibility remain incompletely defined. Current evidence supports a multifactorial model involving autonomic dysregulation, altered vascular responsiveness, neurohumoral influences, and environmental triggers (9–12). However, these factors alone do not adequately explain the familial aggregation observed in some patients, suggesting that genetic predisposition may contribute to disease susceptibility.

In the present pedigree, the heterozygous *WFS1* missense variant c.1480G>A (p.Gly494Ser) showed perfect co-segregation with recurrent VVS across three generations: all affected carriers harbored this variant, whereas genotyped unaffected relatives did not. We emphasize that such familial segregation alone cannot establish causal relationships between this variant and syncopal phenotype.

Several lines of evidence support biological plausibility for this genotype-phenotype association: (1) high evolutionary conservation of Gly494 among vertebrate orthologs; (2) extreme population rarity (max MAF = 0.0004), precluding common benign polymorphism; (3) concordant damaging predictions from PolyPhen-2 and REVEL. SOPMA and AlphaFold simulations predicted minor local conformational perturbations near the substitution site. Of note, these in-silico outputs have inherent limitations for large ER transmembrane proteins and cannot independently verify impaired wolframin stability or protein-protein interactions. All functional inferences are derived exclusively from bioinformatic predictions, without cellular experimental evidence demonstrating that p.Gly494Ser alters wolframin expression, subcellular localization, protein stability, ER homeostasis, calcium handling, insulin signaling or cellular stress responses. Collectively, computational observations generate a testable hypothesis that p.Gly494Ser may exert functional consequences, which awaits dedicated cellular assays.

*WFS1* encodes wolframin, an ER-resident transmembrane protein critical for ER homeostasis, calcium regulation and cellular stress responses (13). Disrupted wolframin function impairs both neuronal and endocrine tissues (14, 15). Highly expressed within hypothalamic and brainstem regions involved in neuroendocrine and autonomic control, loss-of-function *WFS1* variants produce broad neurological, endocrine and autonomic manifestations beyond classic Wolfram-syndrome features (16). Based on published literature, we hypothesize that subtle structural changes induced by p.Gly494Ser might theoretically modify autonomic reflex regulation; however, this mechanistic model remains purely speculative in the absence of patient-derived cellular or heterologous expression data.

Notably, the phenotype observed within this family deviates substantially from typical Wolfram syndrome. Affected subjects exhibited no optic atrophy, hearing loss, diabetes insipidus or progressive neurodegeneration; recurrent VVS constituted the predominant clinical manifestation. This observation expands the phenotypic spectrum linked to heterozygous *WFS1* variants and suggests partial wolframin impairment may give rise to relatively isolated autonomic phenotypes.

*WFS1* also contributes to pancreatic β-cell survival, ER homeostasis and insulin secretion (15). Insulin modulates vascular and autonomic function via endothelial nitric-oxide-dependent pathways (17–19). Excessive nitric-oxide-mediated vasodilation exaggerates orthostatic responses and underlies positive tilt-table outcomes in VVS patients (20, 21). The proband presented with obesity, an established confounder driving peripheral insulin resistance and compensatory pancreatic hyperinsulinemia. We therefore postulate that hyperinsulinemia in the proband arises from combined β-cell dysfunction attributable to the *WFS1* variant and obesity-related insulin resistance.

This study bears several limitations. We did not measure nitric-oxide bioavailability, circulating vascular mediators, baroreflex sensitivity, heart-rate variability or ambulatory autonomic parameters. Without these biomarkers, whether hyperinsulinemia triggers VVS by modifying vasomotor tone or autonomic reflexes cannot be ascertained. Accordingly, hyperinsulinemia observed in the proband and the proband’s mother (the proband’s maternal grandmother lacked insulin-release testing) merely represents an incidental clinical feature co-occurring with the *WFS1* variant, rather than definitive mechanistic evidence for VVS pathogenesis. Prospective studies with comprehensive autonomic-vascular assessments are required to disentangle additive effects of *WFS1* variants, obesity-associated insulin resistance and hyperinsulinemia on VVS susceptibility.

Taken together, our clinical, familial-genetic and bioinformatic findings hypothesize that heterozygous *WFS1* variants exemplified by p.Gly494Ser may act as genetic susceptibility modifiers lowering thresholds for reflex-mediated cardiovascular responses in predisposed individuals, especially upon environmental or physiological triggers. This framework fits the multifactorial nature of VVS and partly accounts for variable expressivity within affected pedigrees; formal functional investigations are required to validate this hypothesis.

### 4.1 Clinical implications

The identification of a co-segregating *WFS1* variant within this single pedigree with recurrent early-onset VVS only indicates that rare genetic variants may contribute to disease susceptibility in a small subset of VVS patients with strong familial clustering, and these findings cannot be generalized to sporadic VVS cases. Routine genetic testing is not recommended for most individuals with VVS; nevertheless, genetic evaluation may be considered for patients exhibiting prominent family history, childhood-onset disease, recurrent syncope, or atypical clinical manifestations. Better recognition of potential genetic predisposition facilitates individualized genetic counseling and long-term clinical surveillance, whereas mechanistic conclusions await future functional validation.

Moreover, the present findings highlight that genes governing ER homeostasis, neuronal signaling and autonomic regulation merit further investigation in pediatric VVS. Expanded genetic studies will improve our understanding of disease heterogeneity and uncover novel biological pathways underlying autonomic dysfunction.

### 4.2 Limitations and future directions

Several important limitations should be acknowledged. First, this study is restricted to one single family and lacks replication in independent cohorts. Second, formal statistical analyses could not be performed due to limited available family members. Third, no functional experiments were conducted; biological effects of p.Gly494Ser are inferred solely from computational predictions. Fourth, additional genetic, epigenetic and environmental contributors to VVS cannot be excluded. Fifth, secondary- and tertiary-structure predictions for wolframin rely entirely on in-silico tools (SOPMA, AlphaFold2), which carry well-known constraints for large multi-pass ER transmembrane proteins. Such computational outputs serve merely as exploratory evidence instead of definitive proof of pathogenic structural defects; follow-up in vitro structural and cellular functional assays are required to confirm whether p.Gly494Ser disrupts wolframin folding and ER homeostasis.

Most critically, this work lacks experimental functional validation. Cellular assays were not performed to assess whether p.Gly494Ser alters wolframin abundance, ER subcellular localization, protein stability, ER-stress signaling, intracellular calcium handling, insulin-associated pathways, or cellular stress responses using patient-derived cells or heterologous expression systems. All functional inferences for this variant originate exclusively from in-silico predictions and published literature; mechanistic interpretations remain speculative without confirmatory laboratory data.

Future work should prioritize functional characterization of this variant, including assessments of ER homeostasis, calcium signaling and neuronal-autonomic function in patient-derived cells or heterologous models. Validation in larger family-based and case-control cohorts is also warranted to determine whether *WFS1* variants are enriched among VVS individuals and clarify their contribution to disease susceptibility.

### 4.3 Conclusions

Within this multigenerational pedigree, the heterozygous *WFS1* variant c.1480G>A (p.Gly494Ser) co-segregates with recurrent vasovagal syncope across three generations. This co-segregation pattern is supported by familial transmission, evolutionary conservation of the altered residue, population rarity, and in-silico structural predictions; nevertheless, these data cannot establish causality between this variant and VVS.

Given the limitation of a single-family design, causal inference remains precluded. Our findings hypothesize that p.Gly494Ser may serve as a candidate susceptibility variant for VVS. This report broadens the phenotypic spectrum linked to heterozygous *WFS1* variants and delivers preliminary hypothesis-generating evidence implicating ER-associated and autonomic regulatory pathways in VVS predisposition. Rigorous replication in large cohorts and comprehensive functional assays are mandatory to validate these observations and define the wider clinical relevance of this variant.

## Data Availability

The original contributions presented in the study are included in the article/Supplementary Material, further inquiries can be directed to the corresponding author (Tables 3, 4).
